# Characterization of the avian influenza viruses distribution in the environment of live poultry market in China, 2019–2023

**DOI:** 10.1186/s40249-025-01304-w

**Published:** 2025-05-09

**Authors:** Hong Bo, Ye Zhang, Jie Dong, Xiyan Li, Xiang Zhao, Hejiang Wei, Zi Li, Dayan Wang

**Affiliations:** 1 National Key Laboratory of Intelligent Tracking and Forecasting for Infectious Disease, National Institute for Viral Disease Control and Prevention, Chinese Center for Disease Control and Prevention, National Health Commission, Beijing, 102206 China; 2 Key Laboratory for Biosafety, National Health Commission, Beijing, 102206 China

**Keywords:** Avian influenza viruses, Subtype, Live poultry market, Environment

## Abstract

**Background:**

The prevalence and transmission of avian influenza viruses (AIVs) in the live poultry market (LPM) is a serious public health concern. This study was to investigate the prevalence of different subtypes of avian influenza viruses in environment of LPM, and to analyze the differences and seasonality of the nucleic acid positive rate (NAPR) of A type, H5, H7, and H9 subtypes in feces, sewage, drinking water, breeding cages, and chopping boards.

**Methods:**

Feces, breeding cages swabs, drinking water, sewage and chopping boards swabs were collected from live poultry market during 2019–2023 from southern and northern China. Real-time PCR was used to screen for virus subtypes. Viruses were isolated, and deep sequencing was performed to obtain whole-genome sequences. Chi-square test was used for statistical analysis of categorical variable, GraphPad Prism software were used to construct graphs.

**Results:**

A total of 64,599 environmental samples were collected from live poultry markets in the southern China and northern China between 2019 and 2023. The average NAPR of the A type was significantly higher in the samples collected from the southern China than in those collected from the northern China (*P* < 0.05). The NAPR of H5, H7, and H9 subtypes carried by the five types of environmental samples in the southern China were significantly different (*P* < 0.05), and a higher NAPR was detected in chopping boards (10.84%), breeding cages (0.28%), and drinking water (40.97%) respectively. The average NAPR of the H9 and H5 subtypes displayed seasonality, reaching a peak in January and February in the southern China, while the peak of the H9 subtype was from October to February in the northern China. A total of 19 subtypes were identified. The H5 subtype significantly decreased, the H7 subtype was almost undetectable, and other subtypes, such as the H3 subtype, increased.

**Conclusions:**

The highly pathogenic H5 subtype has significantly decreased in the live poultry market in China since 2022. However, the proportion of some subtypes, such as the H3 subtype, with low pathogenicity to poultry, has increased, while the H9 subtype remains at a high level. It must be noted that these low pathogenic avian influenza viruses often have no obvious symptoms, can circulate asymptomatically in infected poultry, and are highly pathogenic to humans. Our findings provide insights into the control and prevention of avian influenza viruses and the risk of pandemics associated with avian influenza viruses in the live poultry market.

**Supplementary Information:**

The online version contains supplementary material available at 10.1186/s40249-025-01304-w.

## Background

The prevalence and transmission of avian influenza viruses (AIVs) in the live poultry market (LPM) have been the subject of ongoing global discourse because of their potential implications for zoonotic diseases [1]. LPMs harbor both highly and lowly pathogenic AIVs. Ten hemagglutinin (HA) subtypes of AIVs (H1–H7 and H9–H11) have been previously identified in LPMs [2]. Terrestrial birds (chickens and pigeons) and waterfowl (ducks and geese) sold in LPMs have been found to carry more AIVs, mainly including the H9 and H5 subtypes [3]. LPMs facilitate the viral aggregation, replication, re-assortment, mutation, and spread of avian influenza [4]. Some reports indicate that the dominant subtype of avian flu virus shifts constantly. Between 2014 and 2016, the H9 N2 and H5 N6 subtypes were dominant in the northern China and southernChina, respectively [5]. Since 2016, the H9 N2 subtype has been the most prevalent subtype in poultry of China [6]. LPMs have become important vectors for AIVs.

At least 14 subtypes (H3 N8, H5 N1, H5 N6, H6 N1, H7 N2, H7 N3, H7 N4, H7 N7, H7 N9, H9 N2, H10 N3, H10 N5, H10 N7, and H10 N8) of AIVs have been reported to overcome the species barrier and cause sporadic human infections [7]. Evidence indicates that LPMs play a significant role in human AIVs infections [8]. Since 2003, HPAI H5 N1 has emerged, and studies have reported that LPMs are the main source of H5 N1 infections [9]. Available epidemiological data indicate that exposure to LPMs is related to H7 N9 infections. Exposure to H7 N9-infected poultry at LPMs has been implicated as the main risk factor for human infection [10]. In some cases, people exposed to LPMs can be infected without exhibiting symptoms, leading to seroconversion [11]; for example, the H9 N2, H7 N9, H6 N6, H5 N6, H5 N1, and H3 N8 subtypes have been detected in LPM workers. In China, AIV antibody prevalence in the population exposed to LPMs is correlated with an increased risk of infection with the H7 N9 and H9 N2 subtypes among poultry workers [12, 13]. Therefore, LPMs are an important source of human AIV infections.

The environment of LPMs provides opportunities for close contact between humans and AIVs for the across-species transmission of AIVs. Based on the routine environmental surveillance related to AIVs in LPMs in China, this study focused on studying the distribution of AIVs in the environment of LPMs and provided data for risk assessment. Monitoring the changes in AIV in LPMs is essential for controlling avian influenza outbreaks, reducing economic losses, and preventing the spread of AIV to humans.

## Methods

### Collection of environmental samples from LPMs

From January 2019 to December 2023, 64,599 environmental samples were collected from LPMs in 31 provincial-level administrative divisions (PLADs) in China. Five types of samples were collected, namely, feces, sewage, poultry drinking water, surface swabs for breeding cages, and chopping boards used for slaughtering poultry. Sample collection was performed as previously described [14]. Feces or swab samples were put into 5 ml Hank’s medium containing 0.5% bovine albumin, ampicillin (2 × 10^6^ IU/L), streptomycin (200 mg/L), polymyxin B (2 × 10^6^ IU/L), gentamicin (250 mg/L), mycin (0.5 × 10^6^ IU/L), oxygen hydrochloride foxacin (60 mg/L), and sulfamethoxazole (200 mg/L). For liquid samples (drinking water, sewage), 5-ml liquid was collected. The samples were sent to laboratory within 48 h and stored at 4 ℃. The samples were mixed thoroughly, centrifuged at 3000 rpm for 10 min and the supernatants were collected.

### Subtype identification

Real-time PCR assays for the A type and the H5, H7, and H9 subtypes were performed on all samples, with the pairs of primer and probe sets targeting the matrix and HA gene provided in the Chinese National Influenza Surveillance Guidelines [14]. PCR was carried out using the AgPath-ID™ One-Step RT-PCR Kit (Cat#4387422, Thermo Fisher Scientific Inc., Waltham, MA, USA) according to the manufacturer’s instructions.

### Virus isolation

Virus isolation was performed on influenza A-positive samples detected using real-time PCR. Positive samples of the A type were inoculated into the allantoic cavity of 9-days-old specific pathogen-free chicken embryos (Boehringer Ingelheim Biotech Ltd., China), incubated at 37 °C for 48 h, and chilled at 4 °C overnight. The allantoic fluid was harvested. Hemagglutination assay was performed using 1% turkey red blood cells to detect the harvested viruses according to WHO guidelines [15].

### Genome sequencing

Virus total RNA was extracted using a MagMAX™ CORE Nucleic Acid Purification Kit (cat# A32700, Thermo Fisher Scientific Inc., Waltham, MA, USA). RNA was subjected to reverse transcription and amplification using the SuperScript™ III One-Step RT-PCR System with Platinum™ Taq High Fidelity DNA Polymerase (cat#: 12574035, Thermo Fisher Scientific Inc., Waltham, MA, USA). DNA was purified using a QIAquick 96 PCR Purification Kit (cat# 28183, Qiagen Inc., Hilden, Germany). A DNA library was prepared using the Nextera XT DNA Sample Preparation Kit (cat#FC-131–1096, Illumina Inc., San Diego, CA, USA). Whole-genome sequencing was performed on the Illumina MiniSeq Sequencing System, and the data were analyzed using CLC software.

### Figure drawing

GraphPad Prism 10 software (GraphPad Software Inc., San Diego, CA, USA) was used to construct graphs of AIVs seasonality distribution and subtypes compositions.

### Statistical analysis

STATA 15.0 statistical software (Texas, USA) were used to Chi-square test of categorical variable. When the* P* value was less than 0.05, it was considered to be statistically significant.

## Results

### Nationwide analysis of the nucleic acid positive rate (NAPR) of AIVs by subtype and region

A total of 64,599 samples were collected and tested for influenza A virus, and nucleic acid tests were also carried out for the three subtypes (H5, H7, and H9). The NAPR of the A type in the environment of LPMs nationwide was 36.15%, of which the NAPR of the H9 subtype was the highest (29.74%), the H5 subtype was 6.18%, and the H7 subtype was 0.30%.

The NAPR of the A type influenza virus in the nine PLADs in the southern China was higher than 30%, with an average of 43.56%. Jiangxi, Yunnan, and Guangxi accounted for the top three PLADs, with positive rates of 82.3%, 49.41%, and 45.47%, respectively. The NAPR of the other six PLADs was between 30.52% and 38.02%. In the northern China, only Gansu Province, which is adjacent to Sichuan Province, had a higher NAPR of 28.63%, 11 PLADs were from 3.30 to 16.59%, and three PLADs were less than 0.80% (Table 1).

**Table 1:** The average NAPR of the A type in the environment of LPMs in each provincial-level administrative division (PLAD) located in the southern and northern China respectively. *LPM* Live poultry market, *NAPR* Nucleic acid positive rate

No.	Region	PLADs	NAPR (%)
1	The northern China	Hebei	0
2		Tianjin	0
3		Beijing	0.8
4		Ningxia	3.3
5		Jilin	4.55
6		Qinghai	5.39
7		Heilongjiang	7.05
8		Liaoning	8.64
9		Shaanxi	9.36
10		Inner Mongolia	11.16
11		Henan	12.92
12		Shanxi	15.48
13		Shandong	16.2
14		Xinjiang	16.59
15		Gansu	28.63
16	The southern China	Shanghai	0
17		Hainan	2.8
18		Guizhou	10.69
19		Xizang	11.46
20		Jiangsu	12.7
21		Guangdong	15.88
22		Zhejiang	19.7
23		Fujian	30.52
24		Sichuan	31.32
25		Anhui	31.78
26		Hubei	32.42
27		Chongqing	35.13
28		Hunan	38.02
29		Guangxi	45.47
30		Yunan	49.41
31		Jiangxi	82.3

**Fig. 1:**
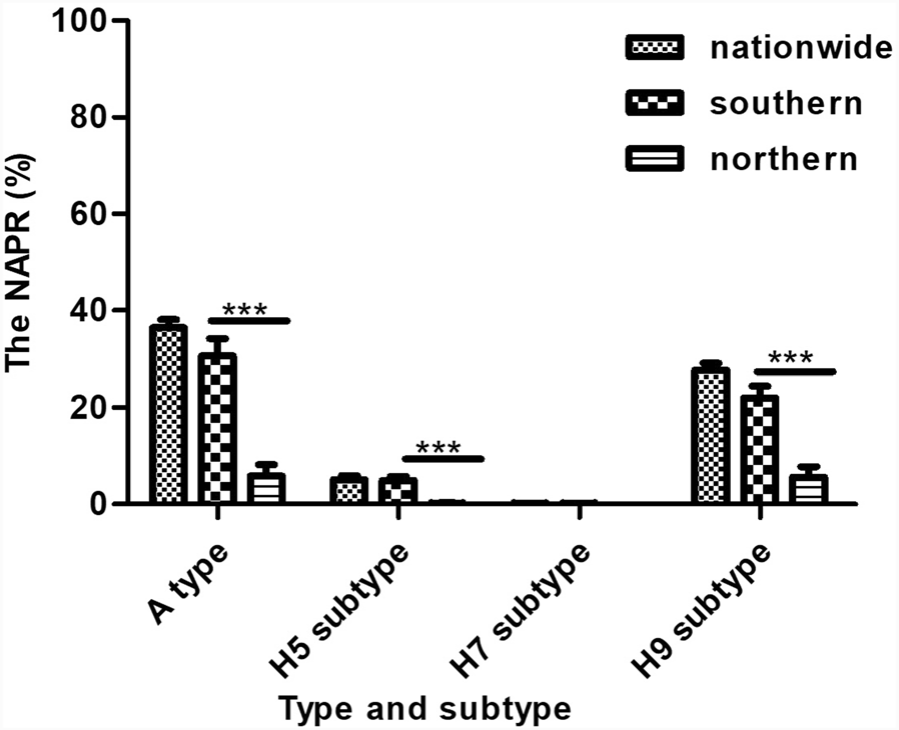
Average NAPR of the A type and the H5, H7, and H9 subtypes in the environment of LPMs of across China and in the southern and northern China. Asterisks (***) represent significant differences at *P* < 0.05. *LPM* Live poultry market, *NAPR* Nucleic acid positive rate

The NAPRs of the H9 and H5 subtypes in PLADs located in the southern China were 33.37% and 7.90%, respectively, which were significantly higher than those of PLADs located in the northern China (*P* < 0.05) (Fig. 1).

### NAPR of AIVs in different types of samples

The NAPRs of AIVs in feces, breeding cages, drinking water, sewage, and chopping boards were analyzed. In the southern China, the NAPRs of the H5, H7, and H9 subtypes were compared, and a significant difference (*P* < 0.05) was found among the different sample types. The NAPRs of the H9 subtype were higher in drinking water and sewage (40.97% and 37.27%, respectively) than in other samples, with less than 8% in breeding cages and chopping boards. The NAPRs of the H5 subtype were significantly higher (*P* < 0.05) in sewage and boards (10.74% and 10.86%, respectively) than in feces, breeding cages, and drinking water. The NAPRs of the H7 subtype were higher in boards and breeding cages (7.68% and 6.46%, respectively).

In northern China, the NAPR of the H9 subtype was the highest (12.9–15.85%) in all five kinds of environmental samples analyzed, and that of the H7 subtype was the lowest (0.05–0.21%). The NAPRs of the H9 and H7 subtypes were statistically analyzed, and no significant differences were observed among the different samples (*P* > 0.05). The NAPR of the H5 subtype was significantly different in the different samples (*P* < 0.05), with 1.30% in feces, which was higher than that of the other sample types (Table 2).

**Table 2:** Nucleic acid positive rates (NAPRs) of the avian influenza virus A type and the H5, H7, and H9 subtypes in five types of environmental samples collected from live poultry markets in the southern and northern China

Region	Sample	NAPR (%) of different subtypes			
		(numbers of positive samples/numbers of samples collected)			
		A type	H5 subtype	H7 subtype	H9 subtype
Southern China*	Feces	36.38	4.40	0.10	26.44
		(4330/11,828)	(521/11,828)	(12/11,828)	(3127/11,828)
	Breeding cages	45.92	6.87	0.28	6.46
		(5339/11,625)	(799/11,625)	(32/11,625)	(751/11,625)
	Drinking water	49.75	6.96	0.14	40.97
		(2080/4181)	(291/4181)	(6/4181)	(1713/4181)
	Sewage	46.85	10.74	0.16	37.27
		(3276/6993)	(751/6993)	(11/6993)	(2606/6993)
	Chopping boards	44.25	10.84	0.27	7.68
		(5604/12,664)	(1374/12,664)	(34/12,664)	(973/12,664)
	*χ*^2^	372.84	449.44	13.27	7200
	*P*	< 0.001	< 0.001	0.01	< 0.001
Northern China*	Feces	14.09	1.30	0.05	12.9
		(522/3704)	(48/3704)	(2/3704)	(478/3704)
	Breeding cages	17.29	0.98	0.07	15.85
		(723/4182)	(41/4182)	(3/4182)	(663/4182)
	Drinking water	15.43	1.22	0.15	14.01
		(316/2048)	(25/2048)	(3/2048)	(287/2048)
	Sewage	15.91	0.55	0.21	14.77
		(461/2898)	(16/2898)	(6/2898)	(428/2898)
	Chopping boards	16.42	1.00	0.13	14.53
		(636/3874)	(39/3874)	(5/3874)	(563/3874)
	*χ*^2^	4.84	10.03	4.30	6.09
	*P*	0.304	0.04	0.37	0.19

* The southern China includes 16 provincial-level administrative divisions (PLADs): Jiangsu, Anhui, Shanghai, Zhejiang, Fujian, Jiangxi, Hubei, Hunan, Guangdong, Guangxi, Hainan, Chongqing, Sichuan, Guizhou, Yunnan, and Xizang. The northern China includes 15 PLADs: Heilongjiang, Jilin, Liaoning, Beijing, Tianjin, Hebei, Shanxi, Inner Mongolia, Xinjiang, Shaanxi, Gansu, Ningxia, Qinghai, Henan, and Shandong

### Seasonality of NAPR of AIV during 2019–2023

**Fig. 2:**
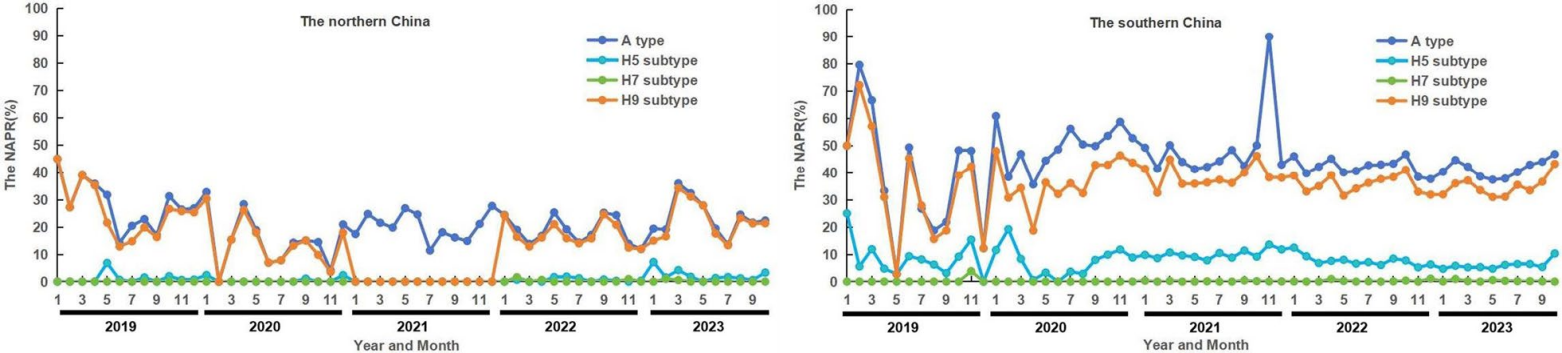
Seasonality of the nucleic acid positive rate (NAPR) of the avian influenza virus A type and the H5, H7, and H9 subtypes in the environment in the northern China and southern China during 2019–2023. Bluish-purple lines represent the A type; blue lines represent the H5 subtype; green lines represent the H7 subtype; and orange lines represent the H9 subtype

In the southern China, the NAPR of the A type peaked from January to February. The NAPR of the H9 subtype peaked between January and February from 2019 to 2020 and in October from 2021 to 2023. The NAPR of the H5 subtype peaked only in January and February from 2019 to 2020, and there was no obvious fluctuation between 2021 and 2023. In the northern China, the NAPRs of the A type and H9 subtype peaked between December and February, and there were no obvious fluctuations in the H5 and H7 subtypes. (Fig. 2 and supplementary 1–2).

### Diversity of influenza subtypes isolated from environmental samples of LPMs

**Fig. 3:**
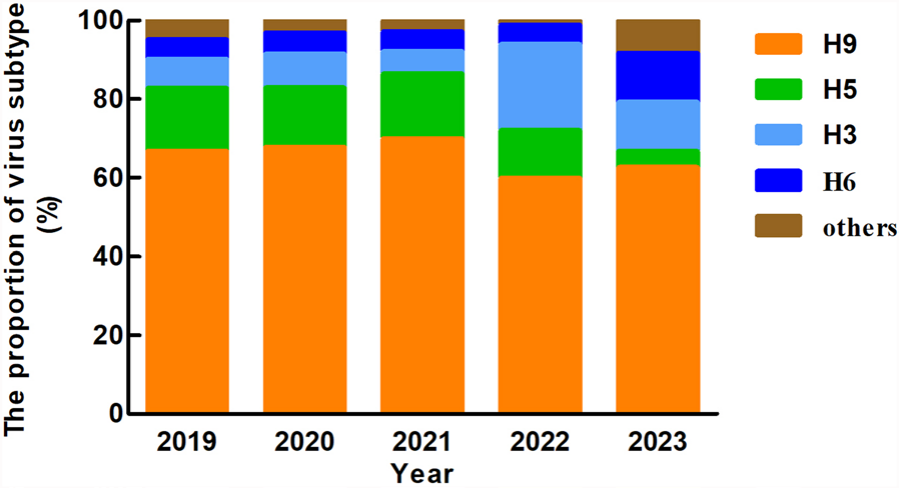
Proportions of the H9, H5, H3, and H6 avian influenza virus subtypes and other subtypes isolated from environmental samples collected from live poultry markets during 2019–2023

A total of 64,599 environmental samples were collected during virus isolation, and 456 AIV strains were obtained. Nineteen subtypes were isolated from the environmental samples collected from LPMs for the period from 2019 and 2023: H1 N1, H1 N2, H1 N3, H2 N3, H3 N2, H3 N3, H3 N8, H4 N2, H4 N6, H4 N9, H5 N1, H5 N6, H5 N8, H6 N6, H7 N9, H9 N2, H10 N3, H11 N2, and H11 N3. Of these subtypes of AIVs, the H9 N2 subtype had the highest proportion, varying between 62.50% and 69.88% during the study period, except for 2022 (40.16%). The proportion of the H5 subtype from 2019 to 2021 was 15.05–16.47%, which decreased significantly to 8.20% and 4.20% in 2022 and 2023, respectively. The proportion of the H3 subtype was 5.68–7.40% between 2019 and 2021 and increased to 21.95% and 12.50% in 2022 and 2023, respectively. The H6 subtype accounts for the highest proportion (12.50%) in 2023 (Fig. 3 and supplementary 3).

For the H5 subtype, three NA subtypes were isolated: N1, N6, and N8. The H5 N6 subtype accounted for the highest proportion of viruses isolated from chopping boards (32.26%), followed by cages (13.58%). The H5 N6 subtype was isolated from four environmental sample types, except for the chopping boards, and the proportions were 1.56–2.78%. The H5 N8 subtype was isolated only from feces and chopping boards. Both the H6 N6 and H3 N2 subtypes accounted for higher proportions in feces (12.50% and 11.11%, respectively) and sewage (9.38% and 15.63%, respectively) (Table 3). The H9 N2 subtype was isolated from five different sample types. The proportion of the H9 N2 subtype was higher than 60% in drinking water (78.43%), breeding cages (69.14%), and sewage (62.50%).

**Table 3:** Subtype proportions of avian influenza viruses isolated from environmental samples

Sample type	Subtype proportion (%) isolated from different sample types								
	H9 N2	H5			H6 N6	H3			Other subtypes*
		N1	N6	N8		N2	N3	N8	
Feces	55.56	2.78	4.17	1.39	12.5	11.11	2.78	1.39	8.33
Breeding cages	69.14	1.23	13.58	0	3.7	4.94	1.23	4.94	1.23
Drinking water	78.43	1.96	7.84	0	3.92	3.92	0	0	3.92
Sewage	62.5	1.56	9.38	0	9.38	15.63	0	1.56	0
Chopping boards	53.23	0	32.26	1.61	3.23	1.61	0	3.23	4.83

*The other subtypes include H1 N1, H1 N2, H1 N3, H2 N3, H4 N2, H4 N6, H4 N9, H7 N9, H10 N3, H11 N2, and H11 N3

## Discussion

Routine AIV-related environmental surveillance has been conducted in China since 2009. Compared with our previous environmental surveillance data of LPMs, the average NAPR of the H9 subtype significantly increased by approximately 27.69%. In addition, virus isolation results indicated that the H9 subtype contributed the highest proportion of subtypes and significantly increased by approximately 10% compared with the period from 2014 to 2018 [16]. The reason for the increase in the H9 subtype might be associated with the source of the collected environmental samples, the poultry supply chain, and the poultry vaccination status. In China, LPMs sell poultry including domestic poultry (chickens), waterfowl (ducks and geese), and rare poultry (quails, pigeons, and partridges). All of these types of poultry can be infected with and carry the H9 N2 virus. Moreover, poultry carrying the H9 N2 virus disseminate the virus through feces and aerosols excreted from the cloaca. In recent years, research has indicated that the prevalence of H9 N2 in poultry in China has shown an upward trend [6]. The poultry sold in LPMs originate from different sources, such as farms and free-range farmers. Some chicken flocks in certain farms are partially immunized or not immunized at all. Previous studies have shown that from 1998 to 2006, the antigenic variation of the H9 N2 virus in poultry did not change significantly, and the vaccination protection rate reached 100% [17, 18]. However, since 2006, the antigenicity of the H9 N2 subtype in poultry changed significantly. In addition, relevant studies demonstrated that vaccination produced the H9 N2 subtype antibody in poultry, HI titer decreased by approximately five times, and multiple gene (HA, NA, PA, PB1, and PB2) mutations were displayed under selection pressure [19–21], resulting in an epidemic strain that cannot be prevented by preimmunized antibodies. H9 N2 subtype evolution and spread were accelerated [22–24]. Therefore, poultry vaccinated with H9 N2 subtype vaccines can still be infected with the H9 N2 subtype in LPMs. In addition, the H5 subtype positive rate has significantly decreased in the past two years, similar to the trend of reduction in human cases; the decline in the NAPR of the H5 and H7 subtypes may depend on H5 + H7 subtype bivalent vaccine usage in poultry [25]. Vaccination has successfully prevented the spread of the H7 N9 subtype in poultry and eliminated human infections with the H7 N9 subtype in China. Poultry are seldom infected by the H7 subtype, reducing environmental contamination.

We also found that the NAPR of the A type was higher in the southern China than in northern China. Moreover, water samples had the highest H9 subtype positivity rates, whereas boards and sewage had higher H5 subtype positivity rates. Several factors may explain these results. Previous studies have reported that live poultry feeding and trading networks are very complex in the southern China [26]. The cities choose their own poultry sales methods according to local conditions, including wholesale markets, wholesalers, retailer markets, and primary and secondary wholesalers [27]. Poultry and meat were sold at LPMs in the southern China. Because of the consumers’ preference for the freshness of live poultry meat, LPMs kept the live poultry selection and provided slaughtering services on-site. In the northern China, there is usually a longer history and habit of purchasing pre-slaughtered poultry meat [28]. Although the Ministry of Agriculture and Rural Affairs of the People's Republic of China has required all PLADs to carry out the 1110 strategy (cleaning every day, disinfecting every week, shutting down once per month, and butchering all unsold live birds before closing every day) since 2018, there are still many kinds of poultry kept together for sale [29].

In our study, water samples (drinking water and sewage) carried more of the H9 subtype, and sewage and chopping boards carried more of the H5 subtype in the southern China. AIVs have been detected in the water, feces, and meat of poultry. Some researchers have reported that virus-contaminated water is an important AIV spreading mechanism among multiple species. Most subtypes (H3 N2, H4 N8, H4 N9, H5 N1, H5 N6, H5 N8, H6 N2, H6 N6, H7 N9, H9 N2, H10 N8, H11 N2, H11 N9, and H12 N7) have been detected in water worldwide [30]. Water from poultry drinking troughs showed the greatest subtype diversity in the LPM. The environmental water used by ducks had the highest number of different subtypes in poultry farms. The LPM, in which different domestic and wild bird species often share the same water, food, and housing, also represents an opportunity for interspecies transmission and viral genetic diversification. Our results also proved that LPM water is the main source of the LPAI H9 subtype and the HPAI H5 subtype.

Notably, the H3 subtype displayed an increasing proportion in the environmental samples, especially the H3 N2 subtype, which was up to 15.63%. It is worth mentioning that the proportion of the H3 subtype is significantly higher than before. In particular, the H3 N8 subtype accounted for 12.19% in 2022. Several factors may have contributed to the increase of the H3 subtype. First, immunization coverage against the H3 subtype is relatively low, leading to the lack of effective protection for poultry flocks. Yang et al. reviewed the evolution of the H3 subtype in China and showed that H3 subtype combinations with multiple NA (N1–N8) subtypes were detected in mainland China and became enzootic in domestic ducks in the southern China [31]. Second, the mixed housing of various types of poultry (chickens, ducks, and wild birds) within LPMs increase the risk of cross-infection [32]. Third, China is located on the East Asia–Australasia Flyway (such as Poyang Lake and Qinghai Lake), and migratory birds (especially waterfowl) may introduce H3 subtype viruses into domestic poultry populations.

Due to differences in live poultry trading policies, local regulations, geographical and ecological conditions, and consumption habits in various regions, there are three limitations in the present study. First, the number of samples of the same type collected from each LPM was inconsistent. Second, the sample types collected from each LPM were not consistent. Third, the species of poultry from which the samples were collected did not remain consistent. These limitations may impact the determination of the NAPR and distribution of AIVs in LPMs in different regions.

## Conclusions

This study suggests that the previously highly pathogenic H5 subtype, which has been alerted for pandemic potential, has significantly decreased in LPMs in China since 2022. However, the proportion of some subtypes, such as the H3 subtype, with low pathogenicity to poultry, has increased, while the H9 subtype remains at a high level. Although these low pathogenic AIVs often cause asymptomatic infections, they can be highly pathogenic to humans, such as the H7 N9 subtype virus. Continuous surveillance and vigilance are needed to prevent the emergence of new reassortant AIV strains and to reduce human infection risks.

## Supplementary Information

Additional file 1.

## Data Availability

The datasets during and/or analysed during the current study available from the corresponding author on reasonable request.

## Abbreviations

AIVs: Avian influenza viruses
LPM: Live poultry market
NAPR: Nucleic acid positive rate
PLAD: Provincial-Level Administrative Division
HA: Hemagglutinin
