# Supplementary material for: Characterization of the avian influenza viruses distribution in the environment of live poultry market in China, 2019–2023
Source: Infect Dis Poverty. 2025 May 9;14:36. doi: 10.1186/s40249-025-01304-w (PMC12063257; doi:10.1186/s40249-025-01304-w)
Supplement: Supplementary file 1 — Additional file 1. [file 40249_2025_1304_MOESM1_ESM.docx]

supplementary1.  **The monthly nucleic acid positivity rate (%) of influenza A, H5, H7 and H9 in the southern China**

| **Month/Year** | **The monthly nucleic acid positivity rate (%)  of A, H5, H7 and H9** | | | |
| --- | --- | --- | --- | --- |
|  | **FluA** | **H5** | **H7** | **H9** |
| Jan-2019 | 50.00 | 25.00 | 0.00 | 50.00 |
| Feb-2019 | 79.63 | 5.56 | 0.00 | 72.22 |
| Mar-2019 | 66.67 | 11.90 | 0.00 | 57.14 |
| Apr-2019 | 33.33 | 4.76 | 0.00 | 30.95 |
| May-2019 | 2.78 | 2.78 | 0.00 | 2.78 |
| Jun-2019 | 49.33 | 9.33 | 0.00 | 45.33 |
| Jul-2019 | 26.74 | 8.14 | 0.00 | 27.91 |
| Aug-2019 | 18.75 | 6.25 | 0.00 | 15.63 |
| Sep-2019 | 21.88 | 3.13 | 0.00 | 18.75 |
| Oct-2019 | 48.28 | 9.20 | 0.00 | 39.08 |
| Nov-2019 | 48.08 | 15.38 | 3.85 | 42.31 |
| Dec-2019 | 12.24 | 0.00 | 0.00 | 12.24 |
| Jan-2020 | 60.88 | 11.56 | 0.00 | 47.96 |
| Feb-2020 | 38.46 | 19.23 | 0.00 | 30.77 |
| Mar-2020 | 46.88 | 8.33 | 0.00 | 34.38 |
| Apr-2020 | 35.67 | 0.58 | 0.00 | 18.71 |
| May-2020 | 44.49 | 3.31 | 0.00 | 36.40 |
| Jun-2020 | 48.55 | 0.00 | 0.00 | 32.15 |
| Jul-2020 | 56.20 | 3.65 | 0.00 | 36.13 |
| Aug-2020 | 50.44 | 2.85 | 0.00 | 32.46 |
| Sep-2020 | 49.83 | 7.97 | 0.00 | 42.86 |
| Oct-2020 | 53.60 | 9.87 | 0.00 | 42.93 |
| Nov-2020 | 58.76 | 11.75 | 0.00 | 46.39 |
| Dec-2020 | 52.75 | 8.82 | 0.00 | 43.73 |
| Jan-2021 | 49.22 | 9.80 | 0.33 | 41.43 |
| Feb-2021 | 41.53 | 8.61 | 0.00 | 32.64 |
| Mar-2021 | 50.16 | 10.70 | 0.24 | 44.89 |
| Apr-2021 | 44.00 | 9.61 | 0.00 | 35.92 |
| May-2021 | 41.31 | 9.05 | 0.00 | 35.95 |
| Jun-2021 | 42.15 | 7.79 | 0.12 | 36.39 |
| Jul-2021 | 44.26 | 10.50 | 0.00 | 37.42 |
| Aug-2021 | 48.34 | 8.77 | 0.00 | 36.26 |
| Sep-2021 | 42.49 | 11.40 | 0.43 | 40.07 |
| Oct-2021 | 50.09 | 9.15 | 0.17 | 46.20 |
| Nov-2021 | 90.05 | 13.59 | 0.07 | 38.30 |
| Dec-2021 | 42.99 | 11.80 | 0.07 | 38.16 |
| Jan-2022 | 46.07 | 12.47 | 0.06 | 38.94 |
| Feb-2022 | 39.78 | 9.30 | 0.00 | 33.06 |
| Mar-2022 | 42.26 | 6.81 | 0.00 | 35.06 |
| Apr-2022 | 45.19 | 7.61 | 0.96 | 39.10 |
| May-2022 | 40.13 | 8.01 | 0.24 | 31.56 |
| Jun-2022 | 40.63 | 6.60 | 0.00 | 34.20 |
| Jul-2022 | 42.80 | 7.15 | 0.10 | 36.27 |
| Aug-2022 | 43.02 | 6.07 | 0.00 | 37.65 |
| Sep-2022 | 43.35 | 8.44 | 0.11 | 38.40 |
| Oct-2022 | 46.80 | 7.77 | 0.39 | 41.07 |
| Nov-2022 | 38.49 | 5.25 | 0.00 | 32.97 |
| Dec-2022 | 37.70 | 6.31 | 1.11 | 31.95 |
| Jan-2023 | 40.35 | 4.70 | 0.07 | 31.97 |
| Feb-2023 | 44.68 | 5.82 | 0.99 | 36.08 |
| Mar-2023 | 42.22 | 5.24 | 0.17 | 37.15 |
| Apr-2023 | 38.60 | 5.31 | 0.00 | 33.55 |
| May-2023 | 37.44 | 4.74 | 0.58 | 31.02 |
| Jun-2023 | 37.87 | 6.16 | 0.29 | 31.14 |
| Jul-2023 | 40.26 | 6.47 | 0.16 | 35.54 |
| Aug-2023 | 42.94 | 6.45 | 0.19 | 33.49 |
| Sep-2023 | 44.07 | 5.35 | 0.10 | 36.67 |
| Oct-2023 | 46.85 | 10.32 | 0.00 | 43.27 |

supplementary**2. The monthly nucleic acid positivity rate (%) of influenza A, H5, H7 and H9 in the northern China**

| **Month/Month** | **The monthly nucleic acid positivity rate (%)  of A, H5, H7 and H9** | | | |
| --- | --- | --- | --- | --- |
|  | **FluA** | **H5** | **H7** | **H9** |
| Jan-2019 | 44.86 | 0.00 | 0.00 | 44.86 |
| Feb-2019 | 27.27 | 0.00 | 0.00 | 27.27 |
| Mar-2019 | 39.02 | 0.00 | 0.00 | 39.02 |
| Apr-2019 | 35.91 | 0.00 | 0.00 | 35.36 |
| May-2019 | 31.82 | 6.82 | 0.00 | 21.59 |
| Jun-2019 | 14.29 | 0.71 | 0.00 | 12.86 |
| Jul-2019 | 20.45 | 0.00 | 0.00 | 14.77 |
| Aug-2019 | 22.90 | 1.53 | 0.00 | 19.85 |
| Sep-2019 | 17.02 | 0.00 | 0.00 | 16.31 |
| Oct-2019 | 31.33 | 2.00 | 0.00 | 26.67 |
| Nov-2019 | 26.45 | 0.65 | 0.00 | 25.81 |
| Dec-2019 | 26.87 | 0.75 | 0.00 | 25.37 |
| Jan-2020 | 32.85 | 2.42 | 0.00 | 30.43 |
| Feb-2020 | 0.00 | 0.00 | 0.00 | 0.00 |
| Mar-2020 | 15.38 | 0.00 | 0.00 | 15.38 |
| Apr-2020 | 28.37 | 0.00 | 0.00 | 26.24 |
| May-2020 | 18.87 | 0.00 | 0.00 | 17.92 |
| Jun-2020 | 6.96 | 0.00 | 0.00 | 6.96 |
| Jul-2020 | 7.81 | 0.00 | 0.00 | 7.81 |
| Aug-2020 | 14.29 | 0.00 | 0.00 | 13.19 |
| Sep-2020 | 15.08 | 1.12 | 0.00 | 15.08 |
| Oct-2020 | 14.49 | 0.00 | 0.00 | 9.81 |
| Nov-2020 | 4.14 | 0.00 | 0.00 | 3.55 |
| Dec-2020 | 20.96 | 2.40 | 0.00 | 17.96 |
| Jan-2021 | 17.43 | 0.00 | 0.00 | 0.00 |
| Feb-2021 | 24.81 | 0.00 | 0.00 | 0.00 |
| Mar-2021 | 21.56 | 0.00 | 0.00 | 0.00 |
| Apr-2021 | 19.80 | 0.00 | 0.00 | 0.00 |
| May-2021 | 26.90 | 0.00 | 0.00 | 0.00 |
| Jun-2021 | 24.60 | 0.00 | 0.00 | 0.00 |
| Jul-2021 | 11.41 | 0.00 | 0.00 | 0.00 |
| Aug-2021 | 18.09 | 0.00 | 0.00 | 0.00 |
| Sep-2021 | 16.18 | 0.00 | 0.00 | 0.00 |
| Oct-2021 | 14.89 | 0.00 | 0.00 | 0.00 |
| Nov-2021 | 21.12 | 0.00 | 0.00 | 0.00 |
| Dec-2021 | 27.75 | 0.00 | 0.00 | 0.00 |
| Jan-2022 | 24.46 | 0.00 | 0.00 | 24.27 |
| Feb-2022 | 18.97 | 0.70 | 1.64 | 16.39 |
| Mar-2022 | 13.81 | 0.00 | 0.00 | 12.86 |
| Apr-2022 | 16.78 | 0.00 | 0.65 | 16.12 |
| May-2022 | 25.36 | 1.67 | 0.00 | 21.05 |
| Jun-2022 | 19.16 | 1.87 | 0.00 | 15.89 |
| Jul-2022 | 14.33 | 1.27 | 0.00 | 14.01 |
| Aug-2022 | 17.09 | 0.00 | 0.00 | 15.82 |
| Sep-2022 | 25.25 | 0.81 | 0.00 | 24.65 |
| Oct-2022 | 24.36 | 0.32 | 0.00 | 20.83 |
| Nov-2022 | 13.88 | 0.00 | 0.96 | 12.44 |
| Dec-2022 | 11.96 | 0.37 | 0.00 | 11.96 |
| Jan-2023 | 19.41 | 7.20 | 0.00 | 15.02 |
| Feb-2023 | 19.13 | 1.53 | 1.02 | 16.58 |
| Mar-2023 | 36.03 | 4.24 | 0.58 | 34.30 |
| Apr-2023 | 32.44 | 1.78 | 0.00 | 31.19 |
| May-2023 | 27.95 | 0.00 | 0.00 | 27.95 |
| Jun-2023 | 19.47 | 1.33 | 0.00 | 17.60 |
| Jul-2023 | 13.60 | 1.70 | 0.00 | 13.31 |
| Aug-2023 | 24.53 | 1.24 | 0.00 | 23.29 |
| Sep-2023 | 21.62 | 0.60 | 0.00 | 21.32 |
| Oct-2023 | 22.39 | 3.31 | 0.00 | 21.37 |

supplementary3. Different subtype of AIVs were isolated and the proportion were calculated during 2019-2020.

| **Subtype** | **Proportion of subtypes (%) during 2019-2023** | | | | |
| --- | --- | --- | --- | --- | --- |
|  | **2019** | **2020** | **2021** | **2022** | **2023** |
| H1N1 | 0.00 | 0.00 | 0.00 | 0.00 | 4.17 |
| H1N2 | 1.23 | 0.00 | 0.00 | 0.00 | 0.00 |
| H1N3 | 0.00 | 0.00 | 0.57 | 0.00 | 0.00 |
| H2N3 | 0.00 | 0.00 | 1.14 | 0.00 | 0.00 |
| H3N2 | 6.17 | 6.45 | 5.68 | 9.76 | 12.50 |
| H3N3 | 1.23 | 2.15 | 0.00 | 0.00 | 0.00 |
| H3N8 | 0.00 | 0.00 | 0.00 | 12.20 | 0.00 |
| H4N2 | 0.00 | 2.15 | 0.57 | 0.00 | 0.00 |
| H4N6 | 0.00 | 2.15 | 0.57 | 0.00 | 0.00 |
| H4N9 | 0.00 | 0.00 | 0.00 | 0.00 | 4.17 |
| H5N1 | 1.23 | 12.90 | 0.00 | 3.66 | 4.17 |
| H5N6 | 14.81 | 5.38 | 14.20 | 8.54 | 0.00 |
| H5N8 | 0.00 | 0.00 | 1.70 | 0.00 | 0.00 |
| H6N6 | 4.94 | 0.00 | 5.11 | 4.88 | 12.50 |
| H7N9 | 1.23 | 67.74 | 0.00 | 0.00 | 0.00 |
| H9N2 | 66.67 | 0.00 | 69.89 | 59.76 | 62.50 |
| H10N3 | 0.00 | 0.00 | 0.00 | 1.22 | 0.00 |
| H11N2 | 1.23 | 1.08 | 0.00 | 0.00 | 0.00 |
| H11N3 | 1.23 | 0.00 | 0.00 | 0.00 | 0.00 |
